# Potential Therapeutic Targets in Uterine Sarcomas

**DOI:** 10.1155/2015/243298

**Published:** 2015-10-21

**Authors:** Tine Cuppens, Sandra Tuyaerts, Frédéric Amant

**Affiliations:** ^1^Department of Oncology, Gynaecologic Oncology, KU Leuven (University of Leuven), 3000 Leuven, Belgium; ^2^Department of Obstetrics and Gynaecology, University Hospitals Leuven, 3000 Leuven, Belgium; ^3^Center for Gynaecologic Oncology Amsterdam (CGOA), Antoni van Leeuwenhoek-Netherlands Cancer Institute, 1066 CX Amsterdam, Netherlands

## Abstract

Uterine sarcomas are rare tumors accounting for 3,4% of all uterine cancers. Even after radical hysterectomy, most patients relapse or present with distant metastases. The very limited clinical benefit of adjuvant cytotoxic treatments is reflected by high mortality rates, emphasizing the need for new treatment strategies. This review summarizes rising potential targets in four distinct subtypes of uterine sarcomas: leiomyosarcoma, low-grade and high-grade endometrial stromal sarcoma, and undifferentiated uterine sarcoma. Based on clinical reports, promising approaches for uterine leiomyosarcoma patients include inhibition of VEGF and mTOR signaling, preferably in combination with other targeted or cytotoxic compounds. Currently, the only targeted therapy approved in leiomyosarcoma patients is pazopanib, a multitargeted inhibitor blocking VEGFR, PDGFR, FGFR, and c-KIT. Additionally, preclinical evidence suggests effect of the inhibition of histone deacetylases, tyrosine kinase receptors, and the mitotic checkpoint protein aurora kinase A. In low-grade endometrial stromal sarcomas, antihormonal therapies including aromatase inhibitors and progestins have proven activity. Other potential targets are PDGFR, VEGFR, and histone deacetylases. In high-grade ESS that carry the YWHAE/FAM22A/B fusion gene, the generated 14-3-3 oncoprotein is a putative target, next to c-KIT and the Wnt pathway. The observation of heterogeneity within uterine sarcoma subtypes warrants a personalized treatment approach.

## 1. Introduction

Although uterine sarcomas only account for 3,4% of all uterine corpus malignancies, they entail a high mortality rate [1, 2]. Reported risk factors are unopposed estrogen stimulation, tamoxifen treatment, obesity, and diabetes [3–5]. However, little is known about their precise etiology, mainly due to their highly divergent genetic aberrations. Together with the rarity of the disease, this contributes to the current lack of optimal treatment modalities. Next to standard hysterectomy (often with bilateral salpingo-oophorectomy), adjuvant treatment options are scarce and depend on the histologic subtype [2]. In this review, we discuss new potential therapeutic approaches in uterine leiomyosarcoma (uLMS), low-grade endometrial stromal sarcomas (LGESS), high-grade endometrial stromal sarcomas (HGESS), and undifferentiated uterine sarcomas (UUS).

## 2. Uterine Leiomyosarcoma

Uterine leiomyosarcomas, arising from the myometrium, are generally high-grade tumors accounting for 60% of all uterine sarcomas [1]. Due to lack of evidence of clinical benefit, adjuvant chemotherapy is not standardly administered in patients with local disease [6]. At least 50% of patients diagnosed with stage I/II uLMS relapse and/or present with distant metastases [7]. For patients with localized metastases, complete metastasectomy enhances disease-specific survival [7]. Adjuvant cytotoxic treatment options are scarce and generally result in limited clinical benefit. The management of advanced uterine LMS has recently been summarized in an extensive review by Amant et al. [8]. The standard first-line treatment consists of doxorubicin ± ifosfamide [8]. The use of gemcitabine ± docetaxel has yielded inconsistent response rates in different studies and is used mostly as a second-line treatment option [9–11]. Interestingly, a randomized phase III study is currently ongoing, assessing the efficacy of gemcitabine + docetaxel, followed by doxorubicin in stage I uterine LMS patients after hysterectomy (ClinicalTrials.gov Identifier: NCT01533207). Another approach in advanced disease is trabectedin, a marine-derived drug that has shown minor first-line and second-line activity in LMS patients, but is currently not approved by the Food and Drug Administration (FDA) [8, 12, 13].

Uterine LMS show multiple and varied genetic aberrations and very complex, often aneuploid or polyploid, karyotypes [14, 15]. This heterogeneity complicates the identification of driver mutations and therapeutic targets. While point mutations are rather scarce in uLMS, its genome is characterized by dispersed large amplifications and deletions, with gains of up to 15% of the genome and losses of up to 45% of the genome [14–16].

### 2.1. Receptor Tyrosine Kinase Signaling

Mutations in receptor tyrosine kinases (RTK), leading to aberrant pathway activation, have often been reported in cancer. Amplifications, mutations, and rearrangements of platelet-derived growth factor (*PDGF*) and its receptor* PDGFR* have been implicated in the pathophysiology of multiple tumor types including gastrointestinal stromal tumor (GIST), glioblastoma, and dermatofibrosarcoma protuberans [17–19]. Although PDGF(R) aberrations have not been studied thoroughly in uLMS, one study reported on PDGFR-*β* amplifications in uLMS [20]. Furthermore, taking together results from three expression studies, 49/215 (23%) uLMS samples (from 128 patients) showed positivity for PDGFR-*β* [7, 21, 22]. Similarly, of 239 uLMS samples retrieved from 128 patients, 108 samples (45%) were moderately to strongly positive for PDGFR-*α*, while no activating mutations have been found in the gene [7, 21, 23]. Despite the finding of strong PDGFR expression in a subgroup of uLMS patients, there are very few reports on targeting this receptor in uLMS. PDGF signaling can be blocked by multitargeted kinase inhibitors, such as imatinib, sunitinib, sorafenib, and pazopanib. Only one report was found on treatment of a uLMS patient with imatinib, which inhibits PDGFR and KIT. The patient was progressive on imatinib treatment and ultimately died due to complications of hypereosinophilia. Sunitinib and sorafenib are multitargeted tyrosine kinase inhibitors that also inhibit vascular endothelial growth factor receptor 2 (VEGFR2) and vascular endothelial growth factor receptor 3 next to PDGFR. Sorafenib is currently used to treat carcinomas but has been tested in a phase II study including LMS patients. However, of 12 uLMS patients, only 4 had stable disease [24]. Sunitinib was suggested by Mahmood et al. to have some activity in LMS but also proved insufficient for treatment of uLMS in a phase II trial by the Gynecologic Oncology Group [25, 26]. Pazopanib inhibits VEGFR, PDGFR, fibroblast growth factor receptor (FGFR), and c-KIT. After a recent successful placebo-controlled phase III trial, the PALETTE study, it was approved by the FDA (April 2012) for use in soft-tissue sarcomas, including leiomyosarcomas [27]. An earlier phase II study reported a progression-free survival rate of 44% at 12 weeks in LMS patients [28].

Although* VEGF* expression in uLMS has been previously explored in IHC studies, results are highly inconsistent. In summary, more than half of the samples (total *n* = 73) were scored positive for VEGF [29–32]. In addition,* VEGFR1* and* VEGFR2* are frequently expressed in uLMS [29, 31]. In case reports, the anti-VEGF monoclonal antibody bevacizumab has resulted in stable disease in one of two uLMS patients [33]. Remarkably, in a case report of epithelioid uLMS, a combination of bevacizumab and the alkylating agent temozolomide resulted in complete remission [34]. Furthermore, a decrease in tumor cell proliferation and angiogenesis and increased apoptosis have been described in* in vitro* and* in vivo* uLMS models after treatment with vandetanib, a VEGFR2/epidermal growth factor receptor (EGFR) inhibitor [35]. Moreover, a recently published phase I study, combining the VEGFR inhibitor cediranib with an inhibitor of *γ*-secretase (an important player in the Notch signaling pathway), reported prolonged stable disease in one of three LMS patients (the responding patient had the uterine subtype; others were not further specified) [36]. In contrast, aflibercept, a VEGF binding and blocking recombinant protein, showed only modest response in a phase II study: 11/41 (27%) uLMS patients had stable disease [37].

Overexpression of* ERBB2/HER-2*, belonging to the ERBB family of tyrosine kinase receptors, is of great clinical importance in breast cancer, where it is tackled by the monoclonal antibody trastuzumab [38]. It has been shown to be amplified in uLMS, but only in one study [20]. Three studies have assessed ERBB2 protein expression in uLMS, with variable results [39–41]. In total, 35 uLMS and 11 uterine sarcomas, not otherwise specified (NOS), were included, of which 12 cases (26%) showed at least moderate staining [39–41]. Hence, a selected group of uLMS patients may benefit from ERBB2 inhibition. However, at present, trastuzumab or other inhibitors have not been tested in uterine sarcomas in preclinical settings, nor in case reports.

Another important cell growth regulator of the ERBB family is* EGFR*. EGFR has been shown to be upregulated in uLMS when compared to normal controls [7]. One study, including 199 tissue microarray samples of 109 uLMS patients, found 72/199 samples to be EGFR-positive [7]. Two other groups compared EGFR expression between uLMS and uterine leiomyomas (LM). Although one group reported significantly increased immunoreactivity in uLMS versus LM, the other group only detected EGFR expression in 1/25 uLMS and in 1/19 LM [23, 31]. A recent report described the activation of the EGFR pathway in uLMS cell cultures, as shown by high receptor phosphorylation levels. Also downstream AKT and mitogen-activated protein kinase (MAPK) pathways were activated, as AKT, EPH receptor B2 (also termed ERK), and ribosomal protein S6 were highly phosphorylated [42]. Interestingly, targeting EGFR with gefitinib rendered uLMS cells sensitive to cytotoxic treatment,* in vitro* as well as in an* in vivo* xenograft model [42].

Insulin-like growth factor 2 (*IGF2*), which activates IGF 1/2 receptors (IGF1/2R), has been reported to be upregulated in uLMS [43, 44]. Some clinical studies using agents that block IGF1R have included LMS patients. A phase II study tested the efficacy of cixutumumab, a selective IGF1R blocker, in sarcoma patients. Only 3/22 (13,6%) LMS patients (NOS) had stable disease at 12 weeks, while other patients were progressive [45]. A phase I study by Macaulay et al. described a partial response in 2/4 LMS patients upon treatment with the anti-IGF1R antibody AVE1642, although patients simultaneously received gemcitabine and erlotinib, an EGFR inhibitor [46]. Another phase I study combined the IGF1R inhibitor figitumumab with the mTOR inhibitor everolimus. A small decrease in tumor size was detected in 1 out of 4 LMS patients (NOS), while another patient had stable disease [47]. Hence, it may be of use to further test combination therapies of IGF receptor blockers with other compounds in uLMS.

Lastly, brain-derived neurotrophic factor (*BDNF*) and its tyrosine kinase receptor, neurotrophic tyrosine kinase receptor type 2 (*NTRK2*, also termed TRKB), have recently been reported to be upregulated in uLMS compared to LM and myometrium. Treatment of MES-SA cells, which are derived from uterine sarcoma, with the multikinase inhibitor K252a or the NTRK2 ectodomain suppressed proliferation and induced apoptosis. Moreover, in MES-SA-injected mouse models, administration of K252a resulted in smaller tumors, lower proliferation rates, and more apoptosis [48].

### 2.2. Upregulated Pathways

**Figure 1:**
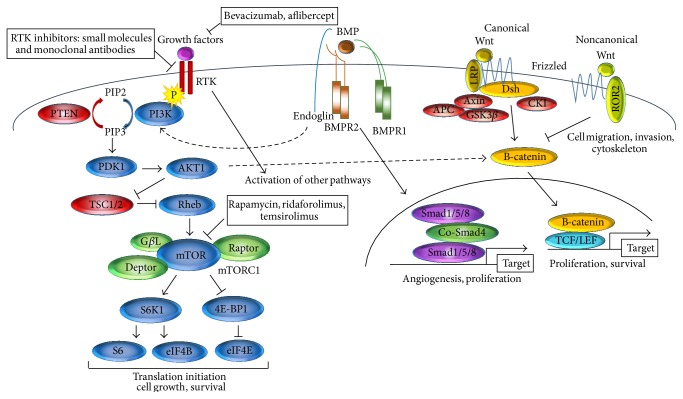
Left: the PI3K/AKT/mTOR pathway is mainly activated by nutrients (not shown) and growth factors, binding to receptor tyrosine kinases and activating PI3K. As PIP2 (phosphatidylinositol 4,5-bisphosphate) is converted to PIP3 (phosphatidylinositol 3,4,5-trisphosphate), PDK1 (pyruvate dehydrogenase kinase, isozyme 1) phosphorylates AKT1 (v-akt murine thymoma viral oncogene homolog 1) upon PIP3-mediated recruitment to the plasma membrane. AKT1 inhibits TSC1/2 (tuberous sclerosis 1/2), relieving the inhibition of Rheb (Ras homolog enriched in brain), which activates mTOR. The recruitment of Raptor (regulatory associated protein of MTOR, complex 1), Deptor (DEP domain containing MTOR-interacting protein), and G*β*L (mLST8; G protein beta subunit-like MTOR associated protein, LST8 homolog) gives rise to the mTOR complex 1 (mTORC1). Upon activation of S6K1 (ribosomal protein S6 kinase 1) and inhibition of 4EBP-1 (EIF4EBP1; eukaryotic translation initiation factor 4E binding protein 1), protein translation is stimulated by activation of ribosomal protein S6 and eIF4B and E (eukaryotic translation initiation factor 4B and E). Additionally, AKT1 activates *β*-catenin signaling. Middle: BMP (bone morphogenetic protein) signaling is modulated through binding of BMPs to BMPR1 and BMPR2 (bone morphogenetic protein receptor type I/II) and the coreceptor endoglin, activating Smad1/5/8 and leading to transcription of target genes involved in angiogenesis and proliferation. Endoglin may also activate PI3K/AKT signaling. Right: canonical Wnt signaling is activated by binding of Wnt to the Frizzled receptor and the LRP (low-density lipoprotein receptor-related protein) coreceptor. Upon recruitment of Dsh (dishevelled) and Axin to the plasma membrane, the *β*-catenin destruction complex, which contains Axin, APC (adenomatosis polyposis coli), GSK3 (glycogen synthase kinase 3), and CKI (casein kinase 1), is inactivated, leading to *β*-catenin accumulation and transcription of target genes after association with TCF/LEF (transcription factor/lymphoid enhancer-binding factor). Noncanonical signaling plays a role in cell migration, invasion, and cytoskeleton arrangement and is mediated through binding of Wnt to Frizzled and other coreceptors such as ROR2 (receptor tyrosine kinase-like orphan receptor 2) or without coreceptors.

Hyperactivation of the phosphatidylinositol 3-kinase/AKT/mammalian target of rapamycin (*PIK3/AKT/mTOR*) pathway has been implicated in uLMS and may play a role in its etiology (Figure 1 displays the discussed pathways and targeted treatments) [49, 50]. The PIK3/AKT/mTOR pathway controls cell growth, proliferation, and survival through regulation of gene transcription and protein synthesis [51]. Phosphorylation of AKT and mTOR has been detected in most uLMS, with phosphorylation of downstream molecules such as eukaryotic translation initiation factor 4E-binding (eIF-4E) and 4E-binding protein 1 (4E-BP1) [49, 52]. Inhibiting the pathway with the natural herb curcumin resulted in apoptosis and reduced cell growth in the uLMS cell lines SKN and SK-UT-1 [53, 54]. These* in vitro* findings were later confirmed* in vivo* [55]. Recently, the clinical response of the rapamycin-analog ridaforolimus was tested in a phase III trial in 711 sarcoma patients, including 231 LMS patients (NOS). While the progression-free survival was modestly increased in patients receiving ridaforolimus, no significant improvement in the overall survival was reached [56]. The FDA did not approve this mTOR inhibitor for the treatment of sarcoma patients, also taking into account its notable toxicity. Another rapamycin-analog, temsirolimus, has led to a partial response (for 17 months) in a uLMS patient, in a phase II trial including 9 LMS patients (NOS) [57]. However, the administration of mTOR inhibitors in combination with a second targeted or cytotoxic agent will likely achieve higher response. For example, combining the mTOR inhibitor rapamycin with the cytotoxic gemcitabine led to cell cycle arrest* in vitro* and this combination was recently confirmed to strongly inhibit tumor growth* in vivo* by an independent group [58, 59]. Furthermore, clinical response was achieved on gemcitabine + rapamycin treatment in an extrauterine LMS patient [60]. After a dose-finding phase I trial for advanced solid tumors, a phase II trial has been completed recently and the results are eagerly awaited [58]. Also, combined targeting of the mTOR pathway and the mitotic checkpoint protein aurora kinase A, using rapamycin + MLN8237, synergistically reduced uLMS cell growth* in vitro*, as well as tumor growth in an* in vivo* model [61].

Next to the mTOR pathway,* Wnt/β-catenin* signaling may be upregulated in uLMS (Figure 1). The Wnt pathway is highly conserved throughout evolution and plays a key role in development [62]. An extensive study by Lusby et al. showed increased expression of *β*-catenin in uLMS compared to normal smooth muscle controls [7]. The authors used 203 samples from 109 uLMS patients and studied *β*-catenin expression in the cytoplasm (low expression in 36% and high expression in 64% of samples) and on the membrane (low expression in 80% and high expression in 20% of samples) [7]. Other groups reported on nuclear expression in 22% and cytoplasmic expression in 87% of 238 uLMS cases [63, 64]. The Wnt/*β*-catenin pathway is targetable through many different pathway players with commercially available inhibitors, but this approach has not yet been tested in uLMS [65].

Moreover, a portion of uLMS tumors are characterized by expression of receptor tyrosine kinase-like orphan receptor 2 (*ROR2*), which is involved in noncanonical Wnt signaling (Figure 1) [62, 66]. ROR2 suppression reduced invasiveness of an LMS cell line* in vitro* and ROR2 knockdown resulted in smaller tumor volumes in xenograft models [66].

In addition to Wnt and mTOR pathways, also transforming growth factor beta/bone morphogenetic protein (*TGF-β/BMP*) signaling may play a role in uLMS (Figure 1). Recently,* endoglin*, a coreceptor in TGF-*β*/BMP signaling, was found to be expressed in 9/22 uLMS. Interestingly,* in vitro* knockdown of endoglin resulted in reduced migration, invasion, and VEGF secretion [67].

### 2.3. Other Targets

In a recent genome-wide study of 12 uLMS, aurora kinase A (*AURKA*) was found to be highly overexpressed. Of note, almost all genes with >9-fold increase in expression were involved in regulating chromosomal homeostasis and spindle assembly, suggesting that proteins involved in these functions could be useful therapeutic targets. Indeed, the single targeting of AURKA with siRNA or MK-5108 inhibited uLMS cell proliferation* in vitro* and decreased the number and size of tumor implants* in vivo* [68]. Similarly, the aurora kinase inhibitor VE465 induced cytotoxicity in the MES-SA uterine sarcoma cell line [69]. Of note, a phase II trial evaluating the AURKA inhibitor alisertib (MLN8237) in pretreated LMS patients has been activated [70].

*MDM2* is an oncogene that negatively regulates p53 function by three mechanisms: (1) targeting p53 for ubiquitin-based degradation, (2) blocking the p53 transcriptional activation domain, and (3) shuttling p53 from the nucleus to the cytoplasm [71]. Blocking MDM2 enhances p53 function and hence provides a therapeutic strategy for many cancer types. Amplifications have been reported in uLMS and in extrauterine LMS [20, 72, 73]. Moreover, MDM2 is overexpressed in 10% of uLMS [74, 75]. MDM2 inhibitors have proven efficient in preclinical settings and, at present, agents such as AMG232 and RG7112 are clinically being explored in various cancer types, although not yet in uterine sarcomas [76–78]. To tackle the problem of resistance to single-agent therapy, Saiki et al. tested potential synergistic combinations of MDM2 antagonists with other compounds* in vitro* in 40 cell lines. Synergy was observed upon simultaneous inhibition of MDM2 and MEK and/or PI3K. Interestingly, this effect was not dependent on the mutation status of genes in the PI3K pathway, and the highest inhibitory effect was noted when all three molecules were blocked [79].

Furthermore, MDM2 inhibitors were found to greatly synergize with histone deacetylase (*HDAC*) inhibitors, as detected by a tremendous increase in apoptosis and decrease in cell proliferation [79]. Histone deacetylases control gene transcription through deacetylation of nucleosomal histones [80]. Of note, HDAC9 was reported to be amplified in 73% of 15 uLMS samples and HDAC8 has been designated as a marker of smooth muscle differentiation and hence may be involved in uLMS, which arises from smooth muscle cells [15, 81]. HDAC inhibition using vorinostat or valproate resulted in growth suppression of the uterine sarcoma cell line MES-SA* in vitro* [69, 82]. Moreover, vorinostat treatment has led to tumor growth reduction of MES-SA-induced tumors* in vivo* [82]. Also, combining vorinostat with the PI3K/AKT/mTOR pathway inhibitors rapamycin or LY294002 showed a synergistic effect on growth inhibition in MES-SA cells [83]. While HDAC inhibitors are clinically being tested in various cancers, mainly in combination regimens with other targeted or cytotoxic treatments, no uLMS patients have been included in these studies to our knowledge [84, 85].

Recently, Edris et al. reported on the use of an antibody against* CD47* for LMS treatment. The antibodies abolish the suppression of phagocytosis that is controlled by macrophage interaction. Interestingly, treatment of uLMS cells with anti-CD47 antibodies increased phagocytosis* in vitro* and reduced uLMS tumor volumes* in vivo* [86].

Further, approximately 50% of uLMS express estrogen receptor (ER) and/or progesterone receptors (PR), [7, 87–90]. A recently published retrospective study on the use of the aromatase inhibitor letrozole in 16 ER/PR positive uLMS patients revealed clinical benefit in 10/16 patients (partial response in 2/16 and stable disease in 8/16 patients). Also the use of the aromatase inhibitor exemestane in second line resulted in clinical benefit in 50% of patients [91]. However, no prospective trials testing hormonal therapy in uLMS have been performed.

### 2.4. Loss of Tumor Suppressor Genes and Synthetic Lethality

In uLMS, recurrent regions of loss often include tumor suppressor genes (TSG) such as phosphatase and tensin homolog (PTEN), tumor protein p53 (TP53), retinoblastoma 1 (RB1), and cyclin-dependent kinase inhibitor 2A (CDKN2A) [15, 92–94].

*CDKN2A* encodes the p16 protein, which controls cell proliferation by inhibiting cell cycle progression. When p16 is absent, cyclin-dependent kinases bind to cyclins, enabling them to phosphorylate RB1. Upon phosphorylation, RB1 releases the transcription factor E2F, stimulating cell cycle progression [94]. In uLMS, several aberrations can alter the cell cycle process. Kawaguchi et al. reported on the inactivation of CDKN2A in soft-tissue LMS by promoter hypermethylation (11/49 cases) and homozygous deletion (3/49 cases). Moreover, 15/49 samples showed decreased p16 expression [94].

Further, the* RB1* gene is frequently deleted in uLMS [15, 93]. The* RB1* gene is named after the corresponding cancer type hereditary retinoblastoma, where the gene is homozygously deleted. It was shown recently that retinoblastoma patients with the hereditary type (RB1 deletion) have an increased risk of uLMS; 3,2% of patients developed uLMS, which corresponds to an excess risk of 3,9/10000 women [95].

*PTEN* is a negative regulator of the PI3K-AKT-mTOR pathway, and loss of PTEN is associated with increased pathway activity, as found in many cancers (Figure 1) [96, 97]. Also in uLMS cells, low levels of PTEN have been associated with high levels of phosphorylated EGFR, AKT, ERK, and S6 ribosomal protein, indicating mTOR pathway activity [42]. Of note, mice that carry homozygous deletions at the* PTEN* locus spontaneously develop LMS, suggesting a strong tumor suppressor role for PTEN in LMS [49]. In preclinical models, loss of PTEN has often been shown to be predictive for response to mTOR pathway inhibition [98–100]. Hence, selection of patients for treatment with PI3K/mTOR inhibitors based on PTEN loss could be a useful strategy in uLMS.

Another tumor suppressor gene, which is mutated in approximately 50% of cancers and frequently deleted in uLMS, is* TP53* [15, 75, 93, 101]. P53 functions in various ways, with major roles in regulating the cell cycle and thus preventing uncontrolled proliferation, and in apoptosis when cells carry highly damaged DNA [101]. Conditional knockout of* TP53* in the reproductive tract of female mice has led to the development of uterine tumors with the uLMS morphology [102]. Additionally, simultaneous loss of the TSG breast cancer 1, early onset (*BRCA1*), and* TP53* accelerated tumor progression in this mouse model. Further, the authors described downregulation of* BRCA1* in 29% of uLMS, most likely due to promoter methylation [102].

Tumor cells that display loss of tumor suppressor function can be tackled by the inhibition of another protein that has become indispensable after loss of the first tumor suppressor, that is, the synthetic lethality principle [103]. For example, inhibition of poly ADP-ribose polymerase (PARP), which is involved in the repair of DNA single-strand breaks, selectively kills cells that are deficient in tumor suppressor protein BRCA1 or BRCA2, which repair double-strand breaks [104]. Similarly, PTEN deficiency has been shown to predict response to PARP inhibitors [105]. However, until present, this approach has not been explored in any uterine sarcoma type. The most important discussed targets in uLMS are displayed in Table 1.

## 3. Low-Grade Endometrial Stromal Sarcoma

Low-grade endometrial stromal sarcomas comprise about 20% of uterine sarcomas [1]. They are myometrium-infiltrating tumors with a notable resemblance to proliferative endometrial stroma [106, 107]. They are often characterized by a less aggressive disease course compared to uLMS, with delayed recurrences [2]. Since LGESS often express ER/PR receptors, patients can benefit from hormonal treatment, for example, by removal of adnexa and progestin therapy, but recurrence rates remain higher than 30% [108, 109]. Unlike uLMS, most LGESS are translocation-related sarcomas [107].

### 3.1. Receptor Tyrosine Kinase Signaling

From IHC studies, the* PDGF* signaling pathway has proven interesting to explore in LGESS. Taking together seven studies, 71/141 (50%) of LGESS cases showed expression of PDGFR-*α* and 53/127 (42%) of LGESS were positive for PDGFR-*β* [21, 110–115]. Interestingly, two case reports of LGESS patients treated with imatinib have shown objective responses [116, 117]. One case was immunohistochemically assessed for the expression of imatinib targets. Whereas the tumor was negative for c-KIT, it was strongly positive for PDGFR-*α* and PDGFR-*β*, designating this receptor as a potential therapeutic target in LGESS and warranting further research [117]. Sardinha et al. investigated* c-KIT* expression in 52 cases and summarized previous studies, revealing only 16 c-KIT positive cases in 203 included LGESS (8% positive), suggesting c-KIT is not a valuable target in LGESS [114]. The only exception is the study by Park et al., where 32/39 LGESS were scored positive for c-KIT [113, 117].

Further, in a total of 156 LGESS samples,* EGFR* expression has been detected in 35 cases (22%), with substantial differences between studies, varying from 0/39 to 14/20 positive cases [110, 111, 113–115, 118, 119]. Hence, the expression and activity of this receptor should be further investigated in LGESS. Overexpression is not the result of gene amplification in LGESS [120]. No studies including LGESS targeting EGFR have been reported to our knowledge.

Combining three studies, with a total of 22 LGESS patients, 60% of tumors expressed* VEGF* [29, 110, 118]. VEGF receptors have not been explored in ESS, except in one study that included 4 samples. All 4 LGESS cases were positive for VEGFR1 and 2/4 tumors showed VEGFR2 staining [29]. A recently published phase I study, combining the VEGFR inhibitor cediranib with a *γ*-secretase inhibitor, reported partial response in a LGESS patient [36]. This promising result warrants further clinical studies with VEGF/VEGFR-targeting agents.

### 3.2. Other Targets

Histone deacetylases and the inhibition thereof are under investigation in many cancer types. In LGESS, overexpression of* HDAC2* has been reported. Although treatment of the ESS-1 cell line with a HDAC inhibitor resulted in cell cycle arrest and cell differentiation* in vitro*, the results failed to translate into clinical response: in a phase II trial exploring the effect of the HDAC inhibitor panobinostat, no responses were observed in 3 LGESS patients [121, 122]. On the other hand, the HDAC inhibitor vorinostat induced cell death via autophagy and it affected mTOR signaling as it reduced mTOR, phospho-S6, p-p70S6K, and p-4E-BP1 levels [83, 123]. Interestingly, further blocking the PI3K/AKT/mTOR pathway using rapamycin or LY294002 in combination with vorinostat showed a synergistic effect on growth inhibition in the ESS-1 cell line [83]. One report by Wu et al. described mTOR expression in 7 of 54 (13%) LGESS samples, but the PI3K/AKT/mTOR pathway is further underexplored in LGESS [124].

Also little is known on the* Wnt/β-catenin* pathway in endometrial stromal tumors. Taking together results from 4 independent studies, 75/121 LGESS show positive *β*-catenin-staining [64, 125–127]. However, expression of cyclin D1, a direct transcriptional target of *β*-catenin that allows cell cycle progression and contributes to cell proliferation, is only rarely detected in LGESS [115, 126, 128].

*Hormone receptors* are expressed in 70–80% of LGESS, with proven therapeutic importance for over a decade [124, 129–132]. Partial responses have been noted in LGESS patients upon treatment with the aromatase inhibitor letrozole and even complete responses have been achieved on treatment with the progestins medroxyprogesterone acetate and megestrol acetate [133–137]. The antiprogesterone mifepristone resulted in stable disease in 1/2 LGESS patients in a phase II trial [138]. Of note, withdrawing estrogen replacement therapy (ERT) and tamoxifen has also resulted in stable disease [137]. Retrospective studies have shown superior survival rates in patients on progestin therapy compared to patients who received other hormonal treatments, pelvic radiation, or no adjuvant treatment [109, 133]. However, due to the rareness of ESS, no prospective randomized placebo-controlled trials have been published on the use of hormone-directed treatment. All targets in LGESS are summarized in Table 1.

## 4. High-Grade Endometrial Stromal Sarcoma and Undifferentiated Uterine Sarcoma

High-grade ESS (HGESS) arise from the endometrial stroma, but unlike LGESS, they show a high-grade round-cell morphology and are clinically more aggressive [107, 139]. They should be distinguished from undifferentiated uterine sarcomas (UUS), which can arise from the myometrium as well as from the endometrium and show no specific differentiation. While being not considered in the WHO 2003 edition, UUS originating in the myometrium most likely represent the previously described “dedifferentiated uLMS,” supporting their inclusion in the latest WHO 2014 classification [107]. Before, HGESS were frequently categorized as “undifferentiated endometrial sarcoma with nuclear uniformity” (UES-U), while most currently termed UUS cases were designated “undifferentiated endometrial sarcoma with nuclear pleomorphism” (UES-P) [107, 130]. Due to the rarity of the disease, no randomized prospective trials have been completed. Although responses have been observed on treatment with gemcitabine/docetaxel or single-agent doxorubicin, the median overall survival is only 11,8 months [140].

### 4.1. The 14-3-3 Oncoprotein

Recently, the translocation t(10;17)(q22;p13) has been described in HGESS [128, 141–145]. The translocation results in the fusion gene YWHAE/FAM22A/B, which gives rise to a* 14-3-3 oncoprotein* [145]. Interestingly, knockdown of the oncoprotein by shRNA or siRNA reduced cell growth and migration in an ESS cell line, defining it as a potential therapeutic target [145]. At present, no small molecule inhibitor has been developed. Interestingly, a strong correlation has been reported between the YWHAE rearrangement and the HGESS (UES-U) morphology, confirming that HGESS and UUS are different entities [146].

### 4.2. Receptor Tyrosine Kinase Signaling

Similar to LGESS,* PDGFR* signaling may be involved in HGESS and UUS. Both PDGFR-*α* and PDGFR-*β* have been detected in 37% of 30 reported samples, warranting studies on its clinical relevance [111, 114, 115].

Only one group has studied* ERBB2* in HGESS/UUS. In a single study by Amant et al., one of four (25%) cases showed amplification and overexpression in the primary and the recurrent tumor [39]. Hence, trastuzumab treatment may be an option for selected patients.* EGFR* overexpression has been reported more frequently in HGESS/UUS. Taken together, 16/33 (48%) cases were EGFR-positive by IHC [111, 114, 115, 119]. A low-level EGFR amplification has only been described in one report. This patient responded temporarily to imatinib, although no c-KIT expression and no genetic aberrations in c-KIT and PDGFR were detected (expression of PDGFR was not assessed) [147]. Another response to imatinib was reported in a patient with* c-KIT* overexpression [148]. C-KIT was recently reported to be overexpressed in 12/12 HGESS carrying the YWHAE/FAM22A/B fusion [149]. Lastly, a phase II clinical trial was recently started by EORTC, testing cabozantinib as a maintenance therapy in high-grade uterine sarcoma patients (ClinicalTrials.gov Identifier: NCT01979393). Cabozantinib is a multikinase inhibitor targeting VEGFR2, c-Met, Ret, Kit, Flt-1/3/4, Tie2, and AXL, which has been approved for treatment of progressive metastatic medullary thyroid cancer [150]. In the current trial, only patients showing response or having stable disease after chemotherapy (doxorubicin ± ifosfamide) are eligible for maintenance treatment with cabozantinib, which will be compared to a placebo arm.

### 4.3. Other Targets

*Cyclin D1* expression has been detected exclusively in UES-U, whereas all reported UES-P cases were negative [126, 146]. Cyclin D1 expression was confirmed specifically in UES that carried the YWHAE/FAM22A/B rearrangement [128]. Hence, cyclin D1 is correlated with the HGESS-subtype carrying the t(10;17) translocation. The presence of cyclin D1 indicates activation of the* Wnt/β-catenin* pathway (Figure 1). Indeed, expression of *β*-catenin has been demonstrated in 6/7 (85%) UES-U cases and only in 2/6 (33%) UES-P cases [130]. Expression of *β*-catenin has been reported in 9/12 additional HGESS/UUS cases, but these were not further classified [125, 151]. As inhibitors for the Wnt/*β*-catenin pathway are available, it may be valuable to test this approach in translocation-related HGESS cases.

UES-U and UES-P also differ in the expression of* sex hormone receptors*. Kurihara et al. found that ER and PR are present in at least half of UES-U cases, while they were not detected in any UES-P cases [130]. The presence of hormone receptors could confer sensitivity to hormonal treatment. The only group reporting on the administration of progestins in a HGESS/UUS patient with weak ER expression, noted a partial response [140]. A summary of all targets is displayed in Table 1.

## 5. Conclusions

In this review, we summarize the latest reported aberrations with potential therapeutic applicability in uterine leiomyosarcomas, low-grade and high-grade endometrial stromal sarcomas, and undifferentiated uterine sarcomas.

Among the scarce clinical reports on targeted treatments in uterine leiomyosarcoma patients, a promising approach involves tackling the PI3K/AKT/mTOR pathway. However, mTOR inhibition leads to activation of AKT through upstream receptor tyrosine kinase signaling, due to interruption of feedback inhibition [152]. Therefore, we propose mTOR inhibition should be considered mainly in combination with other agents. Preclinical responses have been noted upon combination of mTOR pathway inhibition with aurora kinase A inhibitors, MDM2 inhibitors, or histone deacetylase inhibitors [61, 79, 83]. Interestingly, the recently developed small molecule inhibitor CUDC-907, targeting both HDAC and PI3K, shows activity in many human cancer cell lines and xenografts and, additionally, the molecule seems to block treatment escape of cancer cells by also blocking the RAF-MEK-MAPK pathway [153]. Since synergistic effects of blocking HDAC and mTOR have already been described in uterine sarcoma cells, CUDC-907 may be effective in uterine sarcomas [83]. Similarly, another new small molecule inhibitor, CUDC-101, acts on EGFR, ERBB2, and HDAC, which are all potential targets in uterine sarcomas [154]. CUDC-101 has shown strong activity in human cancer cell lines and it also overcomes resistance by simultaneously tackling escape routes [154]. These findings support preclinical research on CUDC-101 in uterine sarcomas.

Another promising approach in uLMS is the interruption of VEGF signaling. Although most responses are published in case reports, sorafenib and aflibercept made it to a phase II trial, resulting in a minor response: stable disease was reached in 4/12 (33%) and 11/41 (27%) uLMS patients, respectively [24, 37]. So far, pazopanib, a multikinase inhibitor targeting VEGFR, PDGFR, FGFR, and c-KIT, is the only FDA-approved targeted treatment in LMS [27]. Combination treatments should be further tested, for example, combination of VEGF(R) inhibition with an EGFR- or *γ*-secretase inhibitor or chemotherapeutics such as temozolomide [34–36].

In low-grade endometrial stromal sarcomas, particularly aromatase inhibitors and progestins have proven their effectiveness. Further, PDGF and VEGF signaling seem to be potential targets in LGESS, but at the moment only case reports have been published. HDAC inhibitors have been shown to be effective* in vitro*, but combination regimens may be necessary to reach efficacy* in vivo*.

High-grade endometrial stromal sarcomas often carry the translocation t(10;17)(q22;p13), giving rise to the fusion gene YWHAE/FAM22A/B [145]. The resulting gene product, a 14-3-3 oncoprotein, was put forward as a therapeutic target when Lee et al. found that its knockdown leads to reduced cell growth and migration in an ESS cell line [145]. 14-3-3 proteins are expressed in all normal cells and they affect signaling pathways, transcription, and survival [145]. Although at present no inhibitors for the oncoprotein are available, this may be a target to look out for in the coming years.

Moreover, Wnt pathway players have been detected specifically in this HGESS subgroup [126, 128, 130, 146]. Hence, the effect of Wnt pathway blocking should be investigated. Also detected in translocation-related HGESS is c-KIT overexpression [149]. Significant tumor regression upon imatinib treatment in a c-KIT-overexpressing HGESS case supports further use in the clinic [148].

Undifferentiated uterine sarcomas appear to be underexplored, as no targets could be identified from literature. Although partly due to their rarity, the recent reclassification of the tumor subtypes by the World Health Organization also impedes the identification of the UUS subtype in older publications [107].

In conclusion, combinations of treatments and multitargeted compounds such as pazopanib overall generate higher clinical benefit than single agents, partly by tackling escape routes that lead to resistance. While multiple promising targets were identified for uterine leiomyosarcoma and low/high-grade endometrial stromal sarcoma, the lack of studies on undifferentiated sarcomas warrants more multicentric studies on these rare tumors. Since randomized trials are scarce for uterine sarcoma patients, we support a personalized therapy approach. Preclinical studies testing the rational combination of existing inhibitors on cell lines and xenografts might pave the way to use these inhibitors in off-label use or repurposed drugs in uterine sarcoma patients, corresponding to their genetic profile.

## Figures and Tables

**Table 1:** Overview of potential therapeutic targets and corresponding treatments in uterine sarcomas.

	Therapeutic target	Targeted agents
uLMS	HER-2	HER-2 inhibitors (e.g., trastuzumab, CP-724714, CUDC-101)
	EGFR	EGFR inhibitors (e.g., gefitinib, erlotinib, cetuximab, vandetanib)
	PDGFR	PDGFR inhibitors (e.g., pazopanib, imatinib, sunitinib, sorafenib)
	VEGF-VEGFR	VEGF-VEGFR inhibitors (e.g., bevacizumab, aflibercept, vandetanib, cediranib)
	IGF1R	Figitumumab, cixutumumab, AVE1642
	BDNF-NTRK2	BDNF-NTRK2 inhibitors (e.g., K252a)
	PIK3/AKT/mTOR Loss of PTEN	PIK3/AKT/mTOR pathway inhibitors (e.g., curcumin, rapamycin, ridaforolimus)
	AURKA	AURKA inhibitors (e.g., MLN8237, MK-5108, VE465)
	Wnt/*β*-catenin	*β*-catenin inhibitors (e.g., LGK-974, PKF118-310, PNU-74654)
	ROR2	ROR2 inhibitors (not yet developed)
	Endoglin/CD105	Anti-CD105 antibodies (in development)
	MDM2	MDM2 inhibitors (e.g., AMG232, RG7112)
	HDAC	HDAC inhibitors (e.g., vorinostat, valproate)
	CD47	Anti-CD47 antibodies (in development)
	ER, PR	Aromatase inhibitors (e.g., letrozole, exemestane) Progestins (e.g., medroxyprogesterone acetate, megestrol acetate)
	Loss of TSG	Synthetic lethality principle (e.g., PARP inhibitors)

LGESS	PDGFR	PDGFR inhibitors (e.g., pazopanib, imatinib, sunitinib, sorafenib)
	EGFR	EGFR inhibitors (e.g., gefitinib, erlotinib, cetuximab, vandetanib)
	VEGF-VEGFR	VEGF-VEGFR inhibitors (e.g., bevacizumab, aflibercept, vandetanib, cediranib)
	HDAC	HDAC inhibitors (e.g., vorinostat, valproate)
	Wnt/*β*-catenin	*β*-catenin inhibitors (e.g. LGK-974, PKF118-310, PNU-74654)
	ER, PR	Aromatase inhibitors (e.g., letrozole) Progestins (e.g., medroxyprogesterone acetate, megestrol acetate)

HGESS	14-3-3 oncoprotein	14-3-3 oncoprotein inhibitors (not yet developed)
	PDGFR	PDGFR inhibitors (e.g., pazopanib, imatinib, sunitinib, sorafenib)
	HER-2	HER-2 inhibitors (e.g., trastuzumab, CP-724714, CUDC-101)
	EGFR	EGFR inhibitors (e.g., gefitinib, erlotinib, cetuximab, vandetanib)
	c-KIT	c-KIT inhibitors (e.g., imatinib, pazopanib)

HGESS/UUS	Tyrosine kinases	Cabozantinib
